# The sonographer’s role in RFA therapy of liver lesions

**DOI:** 10.2349/biij.5.1.e8

**Published:** 2009-01-01

**Authors:** S Mandarano, G Mandarano, JH Sim

**Affiliations:** 1 Department of Radiology, The Alfred Hospital, Melbourne, Australia; 2 School of Medical Sciences, RMIT University, Melbourne, Australia

**Keywords:** Radio frequency ablation, sonographer

## Abstract

Interventional techniques using ultrasound guidance, such as Radio Frequency Ablation (RFA) of liver lesions, are the domain of the radiologist. However, real time ultrasound imaging as performed by the sonographer, is critical in monitoring the successful insertion and placement of the RFA needle. RFA is used to create a localised and controlled application of heat in order to induce necrosis of cells within the liver lesions.

The role of the sonographer is to assist in establishing the criteria for RFA therapy. This includes assessing the liver to establish how easily the lesion can be identified; the size of the lesion; its proximity to large blood vessels and adjacent vital organs and the access route to the lesion itself. In essence, in this discussion, the focus will be on the sonographic techniques in the assessment of the liver prior to RFA and the RFA procedure itself. A brief review of the clinical role that can be provided by Computed Tomography (CT) and Magnetic Resonance Imaging (MRI) is also included.

## INTRODUCTION

An extensive screening program is ideal to ascertain patients’ various aetiologies suspecting hepatic pathology or its progression. Suspicious lesions are followed up by triphasic Computed Tomography (CT) and a treatment plan organised based on the patient’s individual circumstance.

Ultrasound is the ideal imaging modality to perform Radio Frequency Ablation (RFA) on liver lesions in patients for whom surgical resection is either not an option or not the preferred method of treatment. Ultrasound provides real time, non-ionising imaging to the clinician. Ultrasound is also suited for patients with breathing issues or back problems as the patient does not have to lie completely supine as with CT or Magnetic Resonance Imaging (MRI). CT can also be used in conjunction with ultrasound; or in a small number of cases such as advanced cirrhosis, CT may be the preferred modality.

The focus of this article will be on the sonographer’s role in RFA therapy and the technique of RFA in treating liver lesions.

### Selection criteria for RFA

The sonographer assumes an important role in ensuring the RFA selection criteria are adhered to. This section details the factors critical to the success of RFA liver therapy.

#### Patient Co-operation

For those lesions which are not located peripherally in the liver, conscious sedation and analgesia are provided. In such instances, the patient’s ability to suspend respiration when required is of utmost importance. It is possible to perform this procedure under General Anaesthetic (GA) but the ultimate aim is to treat the lesion with minimally invasive techniques.

Liver lesions located peripherally are best treated under GA with an anaesthetist present. RFA of these lesions is considered too painful both during and after the procedure and as such may affect patient’s ability to co-operate.

#### Sonographic Appearance

Solid lesions of any description with a hypoechoic halo have always been regarded as suspicious and have a high association with malignancy. Most commonly these lesions are characteristic of metastases or Hepato Cellular Carcinomas (HCC’s). Although not an absolute indicator of tumour, lesions with a hypoechoic halo often require further investigation and or treatment of some type regardless of the patient’s health or symptoms. Due to the sonographic properties of some lesions which are highly vascularised, they are more likely to appear hyperechoic. Typically the tumours most often treated by RFA are echogenic foci within the liver. These are classic examples for colon metastases as well as neuroendocrine and vascular primaries. HCC’s are also echogenic [1]. These are generalised descriptions of liver lesions typical of HCC’s or Colo Rectal Carcinoma (CRC) metastases; however, these tumours can appear in any combination of the above or appear totally different to these typical descriptions.

#### Nature of the Lesion

Surgical resection of HCC’s detected in their early stage within a relatively healthy liver is the optimal choice. However if the patient is a non-surgical candidate, then RFA is the next most suitable therapy if the patient has normal bilirubin (both conjugated and unconjugated) and has no significant portal hypertension [2]. Liver lesions amenable to RFA treatment can be either primary hepatic lesions such as HCC’s or secondary metastases from CRC. However only those secondary liver metastases with no extrahepatic spread are considered for RFA.

#### Location

The location of liver lesions to be treated affects the success of the RFA procedure. Lesions that are too close to or invading the walls of major vessels within the liver, are unlikely to be treated successfully. This is because the moving blood draws the heat created by RFA away via convection and so it is not possible to ensure that the liver lesion has been coagulated by the heat effectively. Hence, liver lesions that are 3 cm or more in diameter which are abutting vessels will not be treated successfully [2].

Lesions located close to gastric or intestinal walls may endure heat related injury or even perforation if they are located within the ablation zone. If bowel injury is a possibility, an intraperitoneal injection of dextrose to displace bowel can be performed [2]. As the gastric wall is thicker than the intestinal wall, the gastric wall may be more resilient to heating necrosis during RFA treatment [2].

Proximity of the gall bladder within the necrosis zone is also undesirable as irritation of the gallbladder wall causes pain and possibly an iatrogenically induced cholecystitis. Those tumours located close to larger portal triads are also at risk of damaging the nearby bile ducts and causing the patient great pain [3].

Peripherally located liver lesions also require extra care and preparation. At this centre GA is recommended as the pain is too intense under standard sedation and analgesia. Lesions at the dome of the liver have the added complication of potential damage to the diaphragm. The use of an artificial pleural effusion for RFA of these lesions will help to avoid diaphragm damage [3].

#### Size and Number of Lesions

Most radiologists will treat lesions in liver if:

- lesions number less than or no more than four; of which each is 5 cm or smaller, be they primary or secondary hepatic tumours with not extrahepatic masses [3].
- those patients with a background of liver cirrhosis are classified via the Child-Pugh class system. Only lesions in category A or B would be accepted for RFA [4].

If the target tumour is larger than the ablation periphery, overlapping regions must be performed in order to be sure that the entire tumour has been ablated [5].

A successful ablation should achieve 100^o^C within tissue of a 2 cm to 5 cm radius around the needle tip and is held there between 8 minutes to 20 minutes per session [3]. Ablation is required not only for the entire lesion but also a 2 cm cuff of tumour free margin so that any microscopic tumour invasion at the periphery may also be eradicated. The edge of the ablative region can be observed sonographically by echogenic micro bubbles that occur when the tissue is heated by the radio frequency field [3].

Ideally, tumours should be at least 2 cm away from large portal or hepatic vessels to reduce cooling effects and be at least 1 cm away from liver capsule as this can affect adjacent anatomy and be extremely painful. For optimal results, a tumour should be 3 cm in radius or less [3]. This is because the lesion may require overlapping ablations unless it is less than 2 cm in diameter.

## CONTRAINDICATIONS

The following patients are contraindicated for RFA therapy

- Pacemakers (As patients become part of a closed loop electrical circuit, this may adversely affect pacemaker functioning)
- Close proximity to gallbladder
- Close proximity to major bile ducts
- Haemodynamically compromised patients
- Hip replacements (need to put grounding pads elsewhere)
- Child-Pugh Class C Cirrhosis
- Pregnancy [6]

## RFA MECHANISM

Radio frequency (RF) generators operate at 460 kHz with a power setting of between 50 W to 200 W. Once the desired temperature is reached in target tissue, the power is automatically adjusted to a cool down setting as the Watts required to maintain the desired tissue necrotising temperature is reduced. The patient is part of a closed loop circuit including an:

- RF generation
- An electrode needle
- A large dispersive electrode (ground pads)

An alternating current (AC) field in radiofrequency range is thus created within the patient.

Due to the high resistance of tissue when compared with metal electrodes, those ions in target tissue which surround the electrode are highly agitated while they attempt to follow the direction of the alternating current [2]. This agitation causes frictional heat which acts to cause coagulative necrosis when local tissue temperature is between 50^o^C to 100^o^C. The difference between the small surface area of the needle electrode and the large area of the ground pads causes the generated heat to be focussed and concentrated around the needle electrode [2].

Heating the tissue around the needle tip above 100^o^C causes carbonisation or vaporisation of the tissue. The gas then provides insulation against the ablative field that the physician is attempting to establish. The needle tips are internally cooled to avoid tissue vaporisation.

## PATIENT PREPARATION

Patients are given a suitable appointment time allowing for a procedural time of about 2-3 hours duration. The procedure is considered a day procedure and can proceed in one of two ways;

1. conscious sedation (ultrasound or CT machines required)
2. Full GA if lesion is located peripherally or if patient co-operation is in question, (equipment and anaesthetics team required).

The procedure is usually scheduled as a morning procedure. This is so that the day unit can perform 4 hour post procedure observation and the patient can be discharged on the same day. The patient arrives at least 2 hours before the procedure, to be admitted on time and have any blood product if required. A provisional overnight bed is usually considered should complications arise which may require surgical intervention.

Regardless of which pain management technique is used, a full blood (including platelet count) work up is performed the week prior to the procedure. This will allow ample time to order fresh frozen platelets or other blood corrective products and ensure that the patient has ample time on the morning before the procedure to absorb them.

If the patient has had external imaging performed to identify the lesion, those images are made available to the department in the week leading up to the appointment. This is so that the most appropriate preparation can be made. Body habitus may be a factor to determine if CT combined with ultrasound may be required (e.g. hypersthenic patients where the hepatic flexure of the bowel may make it difficult to see lesions even using an intercostal approach with only ultrasound). A cirrhotic liver may make it difficult for ultrasound to resolve posteriorly located lesions and so a combination of ultrasound with CT may be required.

External imaging can be scanned into PACS (Picture Archiving and Communications System) and original images returned to the patient. This allows portability of imaging such that wherever the procedure takes place (ultrasound department with conscious sedation or CT with ultrasound machine combined, angiography or interventional room with ultrasound machine if full GA required) the images can be displayed at a moment’s notice, and the image manipulated as required (e.g. change zoom factor, brightness / contrast etc).

## DAY OF PROCEDURE

On the day of the booked procedure, the sonographer liases with day unit nursing staff in the early morning. As sonographers, we ensure that full blood profile (namely International Normalised Ratio [INR], Partial Thromboplastin Time [PTT], Prothrombin Time [PT] and platelet count) are available and documented and to be within normal limits. Patients who have a background of liver disease may require platelets or other blood corrective factors before the procedure. To ensure the patient meets these criteria we discuss the patient’s preparation needs with nursing staff from day procedure unit. The preparation in addition to blood counts includes ensuring that the patient has a day bed, the patient has been fasting for 6 hours prior to the examination (we recommend nil orally from midnight the night before), the patient has been admitted and all paperwork is in order, the patient has been changed into a hospital gown and is lying on a trolley. We prefer the patient on a trolley as the mattress is firmer (and so there is less of a concavity of the supine position, whereas the softer bed mattresses allow the middle of the patient to be in a slightly kyphotic position). Ease of portability of the trolley is another factor. Once the patient has had the RFA, the patient must remain in supine position for at least 4 hours to minimise any potential bleeding.

If blood products are required for the patient, discussion with the patient’s primary nurse ensures that the product will have been administered and be effective by the time we send for the patient. If possible, the patient should be cannulated with a suitable line for analgesic drug therapy during the procedure.

Once everything is ready we organise a porter to bring the patient on their trolley with their history and full drug chart.

Once in our department, the patient is identified and the sonographer introduces themselves and the patient is reminded in brief of the procedure. We stress that the patient will be bed bound for the procedure and also for 4 hours post procedure. If they need use of toilet facilities before we begin, it is offered.

## RFA PROCEDURE

Ultrasound machines with the option of compound imaging are ideal to better delineate the lesion(s) within the liver. Harmonics may be useful if the lesion to be ablated is not deep; as tumours deep within the liver will not be observed due to the principles of harmonics. Most often, we use a curved 3 MHz to 5 MHz curved array transducer appropriately covered with a sterile cover using aseptic techniques. Standard gel is used for the initial scan but sterile gel is used on the sterile side of the cover during the procedure. In this facility we used either the Phillips IU22 or the ATL 5000. Sometimes due to the heterogenous or fatty nature of the liver to be treated, it was necessary to use the phased array 2 MHz to 5 MHz transducer. All factors are optimised, depth and focal zone set appropriately at or just deep to the area to be investigated. Standard power and contrast settings are used as per typical abdominal set up (1.1 Mechanical Index and approximately 60 decibels respectively). Gain is set adequately to observe the entire liver parenchyma with the Time Gain Compensation (TGC) at a mildly increasing curve toward the posterior edge of liver.

**Figure 1:**
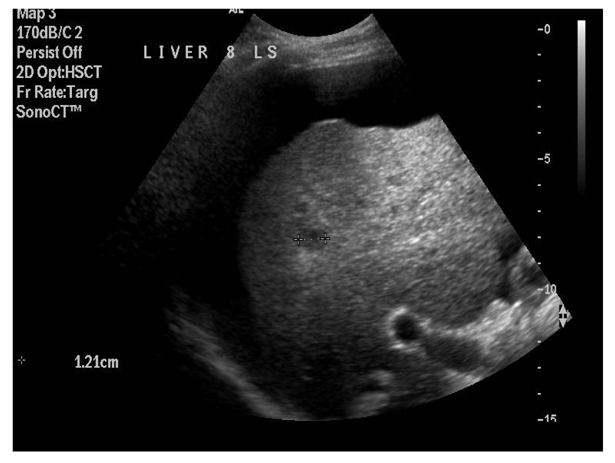
Identification of liver segment and measurement of lesion (prior to RFA).

After observation of previous imaging to identify target lesion, we scan the liver to ensure no further hepatic lesions or biliary pathology are present. The lesion is measured, the liver segment identified and colour Doppler is applied to the B mode image to check vascularity of tumour. In addition, the presence of any major hepatic or portal vessels in the immediate area are identified. If the patient has had recent prior ultrasound imaging in our department we will target our examination to the region of interest. If previous imaging is from an external source or prior imaging is more than a few weeks old, we will perform a full assessment of the liver and observe the gallbladder and bile ducts (figure 1).

Also, as we are scanning we attempt to surmise the method of best approach of needle insertion. Ultimately the radiologist will determine the needle track. However, discussion with the patient may reveal information which will prove valuable in ensuring the success of the procedure. For instance, although a right posterior oblique position – with the patient lying on their left side – using an intercostal approach from lateral side may seem ideal; but if the patient has excruciating left hip pain and cannot lie still regardless of the level of analgesic help, then a more anterior approach could be considered in order to obtain full patient co-operation.

Such information gathered by the sonographer can streamline the preparation and allow the radiologist maximum time for ablation. Also while scanning, the patient is instructed on breathing technique and its importance to the success of the procedure.

Nursing staff are then called in to assemble monitoring devices. A blood pressure cuff and oxygen saturation monitor are attached to the patient. On the opposite arm a needle is inserted (if not already done by day unit nurse) and checked with saline for patency. This line will remain insitu for the duration of the patient’s stay in the hospital for drug administration.

Midazolam and fentanyl are the drugs of choice for sedation and analgesic unless contra-indicated. Oxygen is provided via nasal prongs or mask. The two grounding pads are placed on the patient’s thighs (shaved if necessary). Once an adequate baseline blood pressure and oxygen saturation (finger pulse oxymeter) have been established, the patient is ready to be formally consented in writing by the radiologist. Information gathered by the sonographer is discussed with the radiologist and then the RFA proceeds. The sonographer writes the report and awaits the end of the therapy to finalise the procedure. The radiologist also scans to conclude the best method of approach and practise breathing techniques with the patient.

The skin is prepared after consent and the RFA machine positioned adequately. The grounding pads are also applied.

**Figure 2:**
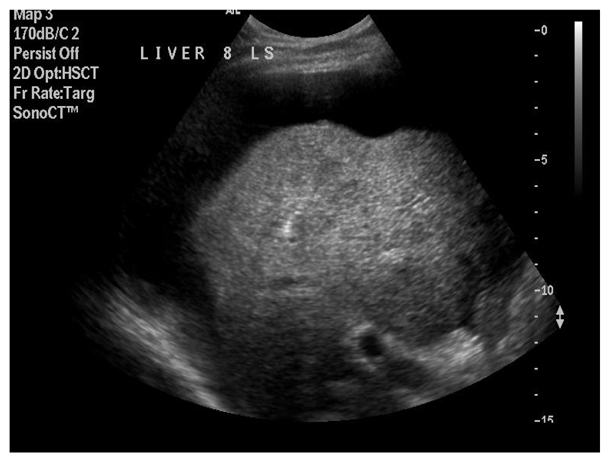
Needle tip (echogenic focus) in centre of lesion to be ablated.

The skin is cleansed with either chlorhexidine or iodine standard solutions using a sterile technique. Ultrasound transducers are covered with sterile covers and sterile gel is used. Using ultrasound guidance the RFA needle is advanced to the target lesion. A plunger at the proximal end of the 15 g needle is advanced [7] to deploy electrodes into the centre of the target lesion (figure 2).

A multi-pronged umbrella of curved electrodes is lodged within the tumour and the generator is switched on. Different types of electrodes (available from a variety of manufacturers) can be used depending on the intended application, lesion size and lesion location.

**Figure 3:**
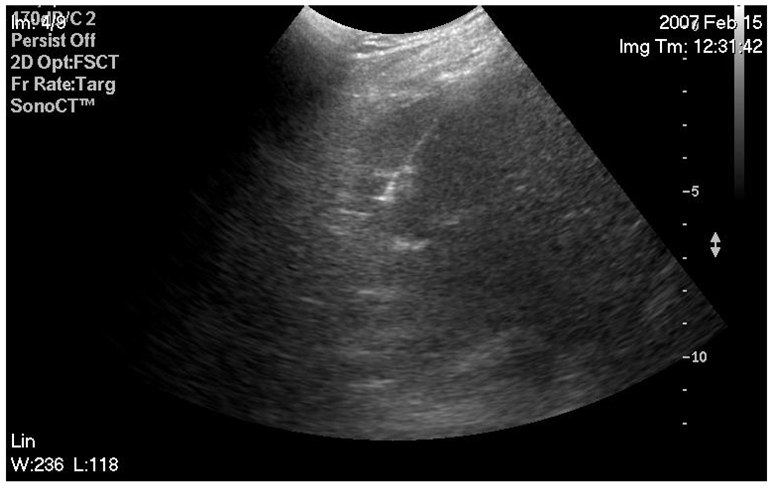
Needle position confirmed immediately prior to ablation.

**Figure 4:**
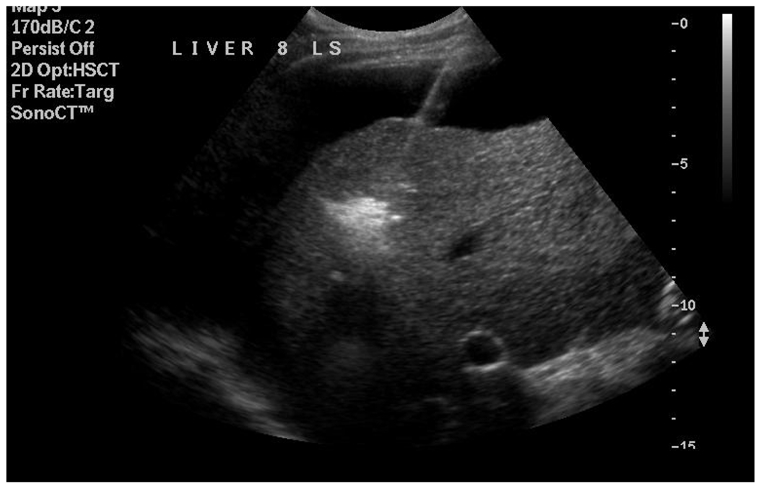
Ablation in progress. Tumour and surrounding tissue are going through tissue necrosis and coagulation. As the tissue is undergoing severe heat conditions, the bleeding vessels are seared and microbubble formation appears as a hyperechoic cloud around the needle tip.

**Figure 5:**
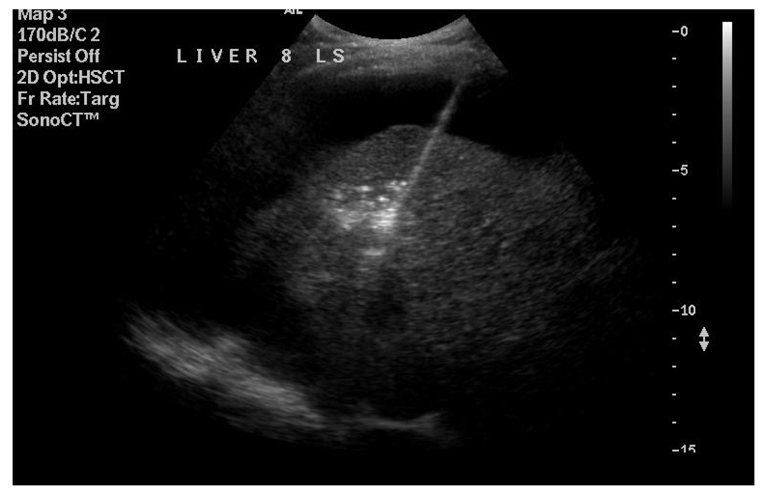
Microbubble formation as tissue necroses. As the ablation continues the microbubbles identify the periphery of the tissue necrosed. This should ideally be an extra 2cm cuff around the visible periphery of the original tumour. The first centimetre to treat non visible spread throughout liver tissue, the next centimetre to treat any microscopic growth outside the original perceived boundary. The hyperechoic cloud recedes and is replaced by a heterogenous, echogenic mass of tissue.

**Figure 6:**
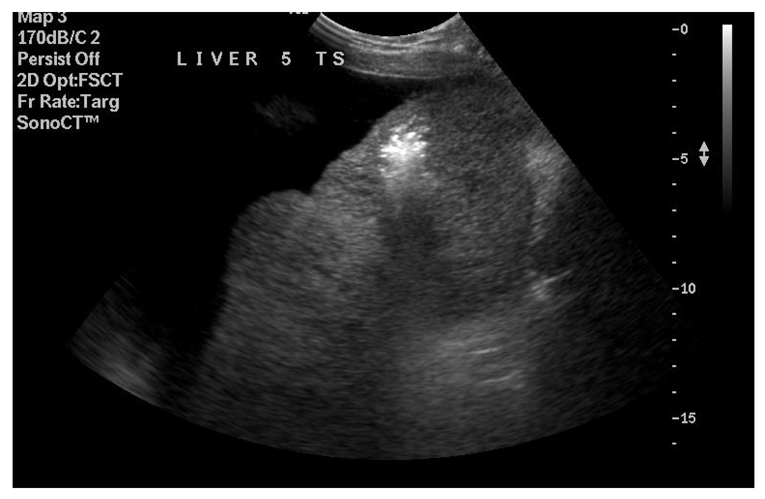
On immediate completion of ablation. With time this lesion will appear less echogenic and shrink in size minimally. Follow up imaging with a triphasic CT or MRI is needed to ensure no tumour was left behind.

The temperature at the tips of the electrodes is controlled to achieve 100^o^C for about 8 minutes to 10 minutes. Once the target temperature is achieved, the system will reduce its output power. The electrodes can then be adjusted (e.g further deployed). The power required to maintain target temperature at the electrode tips is decreased (figures 3-6).

Once the lesion has been ablated, the generator performs a cool down cycle [2]. The electrodes are then retracted and the needle track is also ablated using a lesser temperature of about 70-80^o^C [2] to ensure no seeding of tumour occurs and to coagulate the track to reduce bleeding. Throughout the procedure, the patient’s vital signs are constantly monitored and recorded at various time intervals.

Once the procedure has finished, the day unit nurse is contacted by the radiology nurse for handover to the day unit for observation. The patient is made as comfortable as possible. A suitable dressing is applied to the needle entry site on the skin and the equipment is cleaned and packed away.

## THE ROLE OF CT AND MRI IN RFA

Ultrasound and CT are the two major imaging modalities used to assess liver lesions prior to RFA and CT has been the predominate tool used to assess post RFA treatment success and lesion follow-up [5,8-10].

The real-time capabilities of ultrasound make it the most practical modality to incorporate electrode guidance and intra-procedure assessment [5]. However, both CT and MRI play a role in improving the candidate selection process for RFA and pre-procedure planning [4,11]; such as determining the most appropriate percutaneous pathway [12].

The added advantage of these imaging modalities is that they both provide the benefit of multi-planar imaging – CT via the software reformation process and MRI by direct imaging. Such multi-planar imaging helps to ascertain the appropriate percutaneous pathway. In addition, MRI has the added advantage of superior image contrast capabilities [13].

Traditionally and currently, post procedure follow-up and assessment of tumour ablation success is commonly performed with contrast enhanced CT. However, there is an emerging trend to incorporate MRI into the post-procedure assessment of tumour response to RFA [13]. Some studies are demonstrating that with dedicated open architecture MRI systems, MRI can be used to improve RFA success intra-procedurally [14].The improved intra-procedure success rate and post-procedure monitoring provided with MRI can be attributed to the understanding of the characteristic signal intensity demonstrated by liver tumours and also their contrast media enhancement characteristics. This has been confirmed with histological assessment in one particular study [13].

The MRI signal characteristics can be summarised as follows:

- On non-enhanced images, ablated necrotic tissue is demonstrated as a reduced signal pattern [13-17]. For example, when imaged with MRI, immediately following RF energy application, it is demonstrated as being hypo-intense on a T2 weighted sequence. (CT demonstrates the same ablated necrotic tissue as hypo-dense and ultrasound will display it as hyper-echoic). A relatively hyper-intense rim of 1 to 2 millimetres is also demonstrated. These imaging appearances are in relation to the normal healthy liver parenchyma. The reduced signal intensity is presumed to result from dehydration; that is, water molecules displaced to the periphery of ablation and coagulation zone.
- The zone of reduced signal as described above, should also appear as hypo-intense on a Short Tau Inversion Recovery (STIR) sequence [14].
- Following the administration of intravascular contrast media, a rim or ring enhancement pattern encompassing the ablated necrotic tissue is displayed on a T1 weighted sequence. The necrotic tissue itself should not demonstrate any signal enhancement. This should correspond to the hypo-intense zone demonstrated on the T2 weighted sequence [13-16].
- Histologic assessment of the hyper-intense rim or ring reveals that it is comprised of haemmorhagic components, deteriorating hepatocytes and interstitial oedema [13,14]. This reflects the imaging characteristic of an increased signal.
- The dimensions of induced thermal coagulation, and therefore ultimately tumour necrosis, has been found to have a variable representation across a number of imaging sequences [13,14,16]. Non-enhanced T1 weighted images underestimate this by up to 5 millimetres. STIR images may overestimate this by up to 3 millimetres. T2 weighted images may overestimate by up to 2 millimetres. Contrast enhanced T1 weighted images may overestimate this by up to 4 millimetres.

Intra procedure MRI is not without its own inherent challenges [18]. It adds a further layer to the patient selection process as certain implanted devices are contra-indicated to the MRI environment. Careful attention needs to be paid to the application and placement RF grounding pads and the RFA equipment may induce imaging artefacts which need to be minimised or overcome.

Even though MRI guided ablation adds cost and increased overall procedure time, the benefits of MRI such as direct multi-planar imaging, superior contrast resolution, characteristic signal pattern, ability to better ascertain intra-procedure success, no ionising radiation and accurate post procedure assessment are the factors that render MRI as a very powerful modality to improve patient outcomes and medical management.

## SUMMARY

A sonographer’s expertise is critical in ensuring the success of RFA procedure. However, the sonographer also assumes an important role in the overseeing of staff and equipment coordination with the aim of ensuring the best use of hospital resources. These responsibilities include the coordination of the patient preparation from booking, through to ensuring that blood analysis are performed and if necessary corrected on time, to patient arrival and ensuring the appropriate imaging are available.

## AN EXAMPLE CASE STUDY

The following movie clips (movies 1-3) are taken from the same patient examination. This case is perhaps one of the more difficult types that may be encountered in the clinical setting. It is considered difficult for the following reasons:

- the size of the lesion to be ablated is very small;
- this patient’s liver demonstrates ultrasound image characteristics of advanced cirrhosis, including a heterogeneous texture and irregular contour; and
- the patient has a large body habitus and the lesion under investigation is deep within the liver; and therefore a phased array transducer was used to optimise lesion detection.

**Movie 1:** Pre-RFA: Real-time ultrasound imaging is used to guide the RFA needle to the lesion. Please note the arrow pointing to the needle tip. It appears as a hyper-echoic focus deep within the liver. This is a transverse sector image of the fourth liver segment.

**Movie 2:** RFA procedure in progress: This movie clip demonstrates the RFA process taking place. Note the hyper-echoic foci appearing around the needle tip, indicative of the ablation process.

**Movie 3:** Post RFA: Take note of the hyper-echoic cloud in the region of the ablation. Ultrasound imaging is performed immediately post-RFA to determine that there is no bleeding and that the surrounding blood vessels have not been damaged.
